# Methodological approaches to imputing early-pregnancy weight based on weight measures collected during pregnancy

**DOI:** 10.1186/s12874-021-01210-3

**Published:** 2021-02-05

**Authors:** Jiaxi Yang, Dongqing Wang, Anne Marie Darling, Enju Liu, Nandita Perumal, Wafaie W. Fawzi, Molin Wang

**Affiliations:** 1 Department of Epidemiology, Harvard T.H. Chan School of Public Health, 677 Huntington Ave, Boston, MA 02215 USA; 2 Department of Global Health and Population, Harvard T.H. Chan School of Public Health, Boston, MA USA; 3 Institutional Centers for Clinical and Translational Research, Boston Children’s Hospital, Boston, MA USA; 4 Department of Nutrition, Harvard T. H. Chan School of Public Health, Boston, MA USA; 5 Department of Biostatistics, Harvard T.H. Chan School of Public Health, 677 Huntington Ave, Boston, MA 02215 USA; 6 Channing Division of Network Medicine, Harvard Medical School and Brigham and Women’s Hosptial, Boston, MA USA

**Keywords:** Africa, Tanzania, Pregnancy, Gestational weight, Epidemiologic methods, Statistical model

## Abstract

**Background:**

Early pregnancy weights are needed to quantify gestational weight gain accurately. Different methods have been used in previous studies to impute early-pregnancy weights. However, no studies have systematically compared imputed weight accuracy across different imputation techniques. This study aimed to compare four methodological approaches to imputing early-pregnancy weight, using repeated measures of pregnancy weights collected from two pregnancy cohorts in Tanzania.

**Methods:**

The mean gestational ages at enrollment were 17.8 weeks for Study I and 10.0 weeks for Study II. Given the gestational age distributions at enrollment, early-pregnancy weights were extrapolated for Study I and interpolated for Study II. The four imputation approaches included: (i) simple imputation based on the nearest measure, (ii) simple arithmetic imputation based on the nearest two measures, (iii) mixed-effects models, and (iv) marginal models with generalized estimating equations. For the mixed-effects model and the marginal model with generalized estimating equation methods, imputation accuracy was further compared across varying degrees of model flexibility by fitting splines and polynomial terms. Additional analyses included dropping third-trimester weights, adding covariate to the models, and log-transforming weight before imputation. Mean absolute error was used to quantify imputation accuracy.

**Results:**

Study I included 1472 women with 6272 weight measures; Study II included 2131 individuals with 11,775 weight measures. Among the four imputation approaches, mixed-effects models had the highest accuracy (smallest mean absolute error: 1.99 kg and 1.60 kg for Studies I and II, respectively), while the other three approaches showed similar degrees of accuracy. Depending on the underlying data structure, allowing appropriate degree of model flexibility and dropping remote pregnancy weight measures may further improve the imputation performance.

**Conclusions:**

Mixed-effects models had superior performance in imputing early-pregnancy weight compared to other commonly used strategies.

**Supplementary Information:**

The online version contains supplementary material available at 10.1186/s12874-021-01210-3.

## Background

The role of gestational weight gain (GWG) on pregnancy-related outcomes and future life events for both maternal and child health has been extensively examined [1–9]. In addition, GWG has also been evaluated as an outcome itself with respect to dietary and lifestyle factors [10–12]. GWG is commonly characterized as a single summary measure, such as absolute weight gain during pregnancy or rate of weight gain over a specific time window. Recommendations for GWG have correspondingly been developed using these metrics [13–16].

The use of total weight gain or rate of weight gain to quantify GWG requires the availability of pre-pregnancy weight or at least first-trimester weight (assuming minimal weight gain during the first trimester) [13]. However, this is often challenging, especially in low- and middle-income countries, where few pregnancy cohorts begin maternal weight collection before pregnancy or during the first trimester, as most pregnant women in resource-limited settings do not initiate antenatal care until the second or third trimesters [17]. Consequently, pre-pregnancy or early-pregnancy weights are often unavailable in such studies. Furthermore, even when weights are available during early pregnancy, they are often collected at different gestational weeks, making comparisons of results across different studies difficult.

Various methods have been used in previous studies to impute early-pregnancy weights based on weights collected later during pregnancy [18–20]. To our knowledge, however, no studies have systematically compared the imputation accuracy across different techniques. To fill in this gap with important implication in research implementations, we examined four methodological approaches to impute early-pregnancy weight, including (i) simple imputation based on the nearest one weight measure, (ii) simple arithmetic imputation based on the nearest two weight measures, (iii) mixed-effects models, and (iv) marginal models with generalized estimating equations (GEEs) [21–23]. We used data from two pregnancy cohorts from Tanzania. Because the two studies had different distributions of gestational age at enrollment, they effectively represented two different scenarios where first-trimester weights were either generally available (interpolation) or generally unavailable (extrapolation). We hypothesized that the mixed-effects and GEE models would outperform the two simple imputation approaches. We also hypothesized that weight interpolation would have higher accuracy than weight extrapolation.

## Methods

### Study population

We used data from two randomized, double-blind, placebo-controlled trials conducted in Dar es Salaam, Tanzania. The details of these two studies have been described elsewhere [24, 25]. Briefly, Studies I and II were conducted between 2010 to 2012 and 2010 to 2013, respectively. For both studies, participants were screened and enrolled at antenatal care clinics. Study I enrolled 1500 pregnant women who were randomized to receive a daily oral dose of either 60 mg of iron or placebo from the time of enrollment until delivery [24]. Study II enrolled 2500 pregnant women who were randomized in a two-by-two factorial design to daily oral vitamin A and zinc supplements [25].

At baseline, participants in both studies completed a sociodemographic and reproductive health questionnaire as well as a full clinical examination. They were subsequently followed when the participants were provided with standard prenatal care, and trained research nurses administered health questionnaires and performed an obstetric examination. For our analysis, we excluded participants with missing gestational age at enrollment or multiple fetuses (*n* = 28 for Study I; *n* = 369 for Study II), leaving us with a final sample of 1472 participants for Study I and 2131 participants for Study II.

### Gestational weight assessment

For both studies, weights (kg) at enrollment and monthly follow-up visits were measured by trained study nurses using calibrated scales. Due to the different eligibility criteria, the distributions of gestational age at enrollment differed between the two studies (mean gestational age at enrollment: 17.8 weeks and 10.0 weeks for Study I and Study II, respectively). As a result, the majority of participants in Study I did not have available weight measures collected during the first trimester or early second trimester. In contrast, all of the participants in Study II had at least one weight measure during the first trimester. For each study, implausible weight measures (weight < 30 kg or > 120 kg) were excluded from analysis (number of weight measures: 5 and 27 for Study I and Study II, respectively), leaving us with a total of 6272 and 11,775 available weight measures for analysis from Study I and Study II, respectively.

### Statistical analysis

We evaluated four methodological approaches to imputing early-pregnancy weight in Study I and Study II, separately. Given the timings of available weight measures collected during the follow-up period for each study, we imputed gestational weight at the end of the first trimester, defined as the window between 13 and 15 weeks of gestation. Due to the different distributions of gestational age at enrollment between the two studies, the imputation represented extrapolation (i.e., imputing values farther away from the center of the data range) for Study I and interpolation (i.e., imputing values closer to the center of the data range) for Study II.

To perform weight imputation and evaluate the imputation performance, we divided each study into a testing set and a training set. Training set was used for model development, and testing set was used for model performance evaluation. For the testing set of each study, through simple random sampling, we randomly selected a single sample of 200 participants who had at least one weight measure between 13 and 15 weeks of gestation and at least two weight measures during the entire follow-up period. We chose a sample size of 200 for the testing set based on the small number of participants with available weight measures near the end of the first trimester in Study I (*n* = 231). For women in the testing set with multiple weight measures between 13 and 15 weeks, the measurement closest to 14 weeks and 0 days (i.e., the end of the first trimester) was used as the target time point for imputation. Therefore, the testing set for each study included the weights of the 200 random participants taken at the target time points. These weights were later used as the observed early-pregnancy weights when compared with the imputed weights. On the other hand, the training dataset included all participants and their corresponding weight measurements except the target weight measurements set aside in the testing dataset.

We evaluated the performances of four imputation methods: (i) simple imputation by assigning the nearest weight, (ii) simple arithmetic imputation based on the nearest two weight measures, (iii) mixed-effects models, and (iv) marginal models with generalized estimating equation (GEE). The imputation method assigning the nearest weight measure (method i) was performed by directly taking the weight measure closest to the target time point from the training set as the imputed weight (gestational age of the nearest weight measure: mean [SD] = 18.0 [2.9] and 14.1 [4.1] for Study I and II, respectively). The arithmetic imputation based on the nearest two weight measures (method ii) was performed by identifying the two weight measures closest to the target time point in the training set, calculating the rate of weight gain between the two time points assuming linearity, and then applying the rate to impute the weight at the target time point.

The mixed-effects model method (method iii) was performed by fitting the following mixed-effects regression model for gestational weight in the training dataset:

$$ {W}_{ij}={b}_i+{\beta_i}^Tg\left({t}_{ij}\right)+{\varepsilon}_{ij}, $$

where *W*_*ij*_ represented the *j* th measured weight for the *i* th subject which was measured at gestational week *t*_*ij*_, *g*(*t*_*ij*_) represented a linear or linear plus nonlinear terms of gestational week *t*_*ij*_, *b*_*i*_ and *β*_*i*_ were the subject-specific random intercept and slopes following normal distributions which did not necessarily have zero means, and *ε*_*ij*_ was an error term following a mean-zero normal distribution [18, 26]. The imputed gestational weight for subject *i* at a target gestational week *t* was then ${\hat{b}}_i+{\hat{\beta_i}}^Tg(t).$ Therefore, the between-person variation in gestational weight trajectories was accounted for by including the subject-specific random effects.

The GEE method (method iv) was performed by fitting the following fixed-effects regression model in the training dataset:

$$ {W}_{ij}=\gamma +{\alpha}^Tg\left({t}_{ij}\right)+{e}_{ij}, $$

where *γ* and *α* were the fixed-effects intercept and slopes, and *e*_*ij*_ was a mean-zero error term which was not required to be normally distributed. The imputed gestational weight for subject *i* at a target gestational week *t* was then $\hat{\gamma}+{\hat{\alpha}}^Tg(t)+{\hat{e}}_i$, where ${\hat{e}}_i$ was the average of the residuals, ${\hat{e}}_{ij}$, for the weights at all the gestational weeks available in the training set. Therefore, for the GEE method, the between-person variation in gestational weight trajectories was accounted for by including the subject-specific residuals. We used unstructured variance-covariance matrix for both the mixed-effects model and the GEE methods. Importantly, for both the mixed effects and the GEE methods, the observed weights at the target gestational weeks for which the gestational weights were imputed were not included in the training set in which the regression models were fit.

We evaluated potential non-linear gestational week trajectories by adding quadratic and cubic terms to the model. We also modeled gestational age using restricted cubic splines with three, four, and five knots placed at equally spaced percentiles of the observed gestational weeks in the training set [26, 27]. We additionally explored alternative knot placements with three knots at the 5th, 50th, and 95th percentiles, four knots at the 5th, 35th, 65th, and 95th percentiles, and five knots at the 5th, 27.5th, 50th, 72.5th, and 95th percentiles [18, 26]. For the GEE method, in addition to the mean residual approach described above, we also implemented a nearest residual approach; that was, the imputed gestational weight for subject *i* at the target gestational week *t* was $\hat{\gamma}+{\hat{\alpha}}^Tg(t)+{\hat{e}}_{i{j}^{\prime }},$ where ${\hat{e}}_{i{j}^{\prime }}$ was the residual corresponding to subject *i* ’s measurement in the training set that was closest to the target time *t*.

Using the modeling methods described above, we imputed a subject-specific weight at the target gestational week for each subject in the testing set, who had available weight measurement between 13 and 15 weeks of gestation. Model performance was evaluated based on the mean absolute error (MAE, kg), which was calculated by taking the average of the absolute differences between the imputed weight and the observed weight at the same time point during the pregnancy over the subjects in the testing set. Mean square error (MSE), spearman correlation coefficient (*r*), and proportion of subjects in the testing set with difference in imputed weight and observed weight within 2 kg were also evaluated [16].

Sensitivity analyses included 1) examining the influences of distant weight measures by dropping the third-trimester weights from analysis; 2) including gravidity, age, and education status as predictors in the models; and 3) natural log-transforming weight before fitting the models. All analyses were conducted using SAS statistical software (version 9.4; SAS Institute Inc., Cary, NC, USA). Sample SAS programs are available upon request.

## Results

Study I had 1472 subjects with 6272 observed weight measures; Study II had 2131 subjects with 11,775 observed weight measures. The population characteristics of the studies were summarized in Table 1. The mean baseline gestational age was 17.8 weeks (SD = 4.4 weeks) for Study I and 10.0 weeks (SD = 2.4 weeks) for Study II. The median for the total numbers of weight measurements was 5 (range: 1–9) for Study I and 6 for Study II (range: 1–10). The characteristics of the subjects included in the testing sets were similar to those in the entire datasets for both studies. To visualize the data, we randomly selected 20 subjects from each study and plotted the observed weight measures (Supplement Figs. 1 and 2). Subjects from both studies showed increased gestational weight over the course of pregnancy.

**Table 1:** Population Characteristics of Study I (2010–2012) and Study II (2010–2013), Dar es Salaam, Tanzania

Characteristic	Study I Entire set (***N***=1472)	Study I Testing set (***N***=200)	*p*-value^a^	Study II Entire set (***N***=2131)	Study II Testing set (***N***=200)	*p*-value^a^
Age at baseline (years), mean (SD)	23.9 (4.1)	23.7 (4.0)	0.52	22.6 (3.9)	22.7 (3.8)	0.98
Weight at baseline (kg), mean (SD)	59.7 (11.7)	58.6 (11.7)	0.14	55.6 (11.0)	55.1 (10.1)	0.57
Height at baseline (cm), mean (SD)	156.1 (6.1)	157.1 (6.4)	0.07	154 .7 (6.1)	154.8 (6.1)	0.90
Gestational week at baseline (weeks), mean (SD)	17.8 (4.4)	12.7 (2.1)	< 0.0001	10.0 (2.4)	9.8 (2.4)	0.22
Total number of antenatal visits, range (median)	1–9 (5)	2–9 (6)	< 0.0001	1–10 (6)	3–10 (7)	< 0.0001
Weight at the end of 1st trimester (kg), mean (SD)^b^		58.7 (11.6)			55.3 (9.9)	
Last available weight measure at the end of 2nd trimester (kg), mean (SD)	62.2 (11.7)	62.7 (11.5)	0.58	59.8 (10.7)	59.6 (9.6)	0.82
BMI based on last available weight at the end of 2nd trimester (kg/m^2^), mean (SD)	25.5 (4.6)	25.4 (4.7)	0.70	25.0 (4.2)	24.9 (3.8)	0.68
Gestational age at delivery (weeks), mean (SD)	39.5 (3.5)	39.0 (3.2)	0.07	38.8 (2.7)	39.1 (2.4)	0.084
Treatment status (control), n (%)^c^	738 (50.1)	102 (51.0)	0.79	533 (25.0)	41 (20.5)	0.19
Primigravida, n (%)	613 (41.6)	91 (45.5)	0.23	1024 (48.1)	104 (52.0)	0.25
Marital Status, n (%)			0.36			0.15
Married or co-habiting	1172 (79.6)	164 (82.0)		1908 (89.5)	185 (92.5)	
Other/missing	300 (20.4)^d^	36 (18.0)		223 (10.5)	15 (7.5)	
BMI at baseline (kg/m^2^), n (%)			0.04			0.74
Underweight (< 18.5)	68 (4.6)	16 (8.0)		244 (11.5)	19 (9.5)	
Normal (18.5–25)	840 (57.1)	119 (59.5)		1297 (60.9)	128 (64.0)	
Overweight (25–30)	396 (26.9)	41 (20.5)		420 (19.7)	37 (18.5)	
Obese (≥30)	168 (11.4)	24 (12.0)		169 (7.9)	16 (8.0)	
Education status, n (%)			0.34			0.38
0–4 years	32 (2.2)	5 (2.5)		177 (8.3)	12 (6.0)	
5–7 years	781 (53.1)	104 (52)		1345 (63.2)	133 (66.5)	
8–11 years	406 (27.6)	57 (28.5)		498 (23.4)	42 (21.0)	
≥12 years	214 (14.5)	32 (16.0)		110 (5.2)	13 (6.5)	
Unknown	39 (2.7)	2 (1.0)		1 (0.05)	0 (0.0)	
Occupation status, n (%)			0.69			0.12
Unemployed	700 (47.6)	98 (49.0)		1174 (55.1)	110 (55.0)	
Unskilled or informal	445 (30.2)	63 (31.5)		514 (24.1)	51 (25.5)	
Skilled	280 (19.0)	34 (17.0)		113 (5.3)	4 (2.0)	
Other/unknown	47 (3.2)	5 (2.5)		330 (15.5)	35 (17.5)	
Non-live birth in previous pregnancy, n (%)^e^	126 (20.6)	27 (29.7)	0.02	219 (20.7)	15 (16.7)	0.32
Prior history of complications, n (%)^f^	109 (7.4)	18 (9.0)	0.35	115 (5.4)	7 (3.5)	0.21

*Abbreviations*: *BMI* body mass index

^a^*P*-value from chi-square test comparing categorical covariate and analysis of variance comparing continuous covariate between testing set and the rest of entire set was presented

^b^Among participants with available weight measures taken at end of trimester 1 during 12–14 weeks of gestation who were included in the testing sets

^c^Treatment was 60 mg iron supplement for Study I; Zinc and Vitamin A (as a 2-by-2 factorial design) for Study II (vitamin A only, zinc only, vitamin A and zinc, placebo). N (%) of the control group was presented for each study

^d^One person had missing marital status in Study I

^e^Non-live birth included fetal death, abortion, miscarriage, ectopic pregnancy among non-primigravida women

^f^Prior history of complication included any history of the following: CVD, high blood pressure, diabetes, weight loss in previous year, or ever having a low birth weight baby if non-primigravida

### Weight extrapolation in study I

In Study I, which had fewer weight measures collected during the first trimester compared to Study II, we extrapolated early-pregnancy weight based on weights collected later in the pregnancy. Across the four methods evaluated, the mixed-effects model had the highest imputation accuracy (restricted cubic splines model with three knots at quartiles: MAE = 1.99 kg (SD = 1.70 kg, interquartile range: 0.70–2.65 kg)) (Table 2). Results from the MSE, the correlation coefficient, and the proportion of subjects with difference in imputed weight and observed weight within 2 kg were consistent with the MAE results (the mixed-effects model with the lowest MAE: MSE = 6.86 kg, correlation coefficient = 0.96, proportion of subjects in the testing set with the weight difference within 2 kg = 62%). Varying model flexibility in the mixed-effects model by adding additional polynomial terms or spline terms did not considerably improve the accuracy. Among the other three imputation methods in imputing early-pregnancy weight (assigning the nearest measure, arithmetic calculation using nearest two measures, and GEE method), assigning to the nearest weight measure gave the smallest MAE (nearest weight method: MAE = 2.46 kg; arithmetic calculation using nearest two measures: MAE = 2.91 kg; GEE method with cubic polynomials: MAE = 2.93 kg) (Table 2).

**Table 2:** Results of extrapolating early-pregnancy weights in Study I and interpolating early-pregnancy weights in Study II

Imputation method	Mean Absolute Error (kg)			
	Study I weight extrapolation *N*=1472		Study II weight interpolation *N*=2131	
Mixed-effects models	All weights included (*n*=6272)	Dropping third trimester weights (*n*=3375)	All weights included (*n*=11,775)	Dropping third trimester weights (*n*=8125)
3 knots (quartiles)	1.99	2.01	1.69	1.64
4 knots (quintiles)	2.08	2.05	1.66	1.59
5 knots (sextiles)	2.18	N/A^a^	1.60	1.70
3 knots (5th, 50th, 95th)	2.00	2.00	1.67	1.63
4 knots (5th, 35th, 65th, 95th)	2.00	1.98	1.62	1.66
5 knots (5th, 27.5th, 50th, 72.5th, 95th)	2.25	1.98	1.60	1.81
Linear	2.02	2.01	1.67	1.65
Quadratic	2.02	1.95	1.66	1.62
Cubic	2.02	6.46	1.62	N/A^a^
Marginal models with GEE	Mean residual	Nearest weight residual	Mean residual	Nearest weight residual
3 knots (quartiles)	2.94	3.94	2.03	1.98
4 knots (quintiles)	2.94	3.94	2.00	1.97
5 knots (sextiles)	2.94	3.94	1.96	1.96
3 knots (5th, 50th, 95th)	2.94	3.94	2.02	1.97
4 knots (5th, 35th, 65th, 95th)	2.94	3.93	1.97	1.96
5 knots (5th, 27.5th, 50th, 72.5th, 95th)	2.94	3.92	1.95	2.01
Linear	2.94	3.94	2.01	2.03
Quadratic	2.94	3.94	2.02	1.98
Cubic	2.93	3.92	1.97	1.96
Assigning the nearest weight measure	2.46		2.14	
Arithmetic imputation using the nearest two weight measures	2.91		2.00	

*Abbreviations*: *GEE* generalized estimating equation

^a^Model failed to converge

In the sensitivity analyses, dropping third-trimester pregnancy weights from the mixed-effects models slightly improved the accuracy (Table 2). For the GEE approach, the models with the mean weight residual produced consistently lower MAEs, compared to the models with the nearest weight residual (Table 2). Log-transforming weight or including gravidity, age, or education status as predictors did not improve the accuracy (results not shown).

### Weight interpolation in study II

In Study II, because all women had at least one weight measure collected during the first trimester, we interpolated early-pregnancy weight based on weights collected throughout the pregnancy. Mixed-effects model showed the highest imputation accuracy (restricted cubic splines model with five knots placed at the 5th, 27.5th, 50th, 72.5th, 95th percentiles: MAE = 1.60 kg (SD = 1.72 kg, interquartile range: 0.60–1.20 kg), MSE = 5.49 kg, correlation coefficient = 0.96, proportion of subjects in the testing set with the weight difference within 2 kg = 77%; the sextiles methods had similar results). A slight improvement in accuracy was seen with varying model flexibility in the mixed-effects models. The other three imputation approaches showed similar degrees of accuracy, which were all lower than those from the mixed-effects models (nearest weight method: MAE = 2.14 kg; arithmetic calculation using nearest two measures: MAE = 2.00 kg; GEE method with five knots: MAE = 1.95 kg) (Table 2).

In the sensitivity analyses, we did not observe a consistent pattern of improvement in the weight interpolation analyses when dropping the third-trimester weights (Table 2). GEE methods with the mean residual and the nearest weight residual performed similarly. Finally, log-transforming or including a third covariate did not improve accuracy (results not shown).

**Fig. 1:**
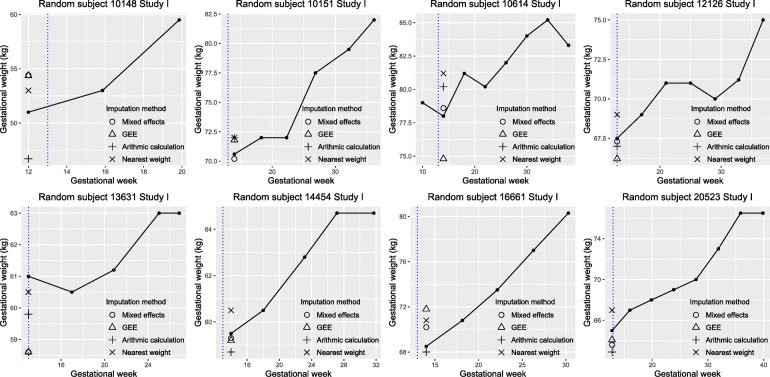
Imputed weights vs. observed weights (kg) of eight randomly selected subjects from Study I testing set based on the four different imputation methods (assigning the nearest weight measure, arithmetic imputation using the nearest two weight measures, mixed-effects model with the lowest mean absolute error, generalized estimating equation (GEE) model with the lowest mean absolute error), Dar es Salaam, Tanzania, 2010–2012

**Fig. 2:**
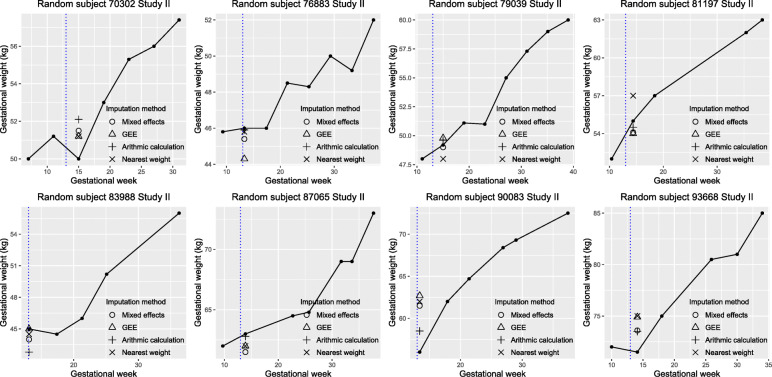
Imputed weights vs. observed weights (kg) of eight randomly selected subjects from Study II testing set based on the four different imputation methods (assigning the nearest weight measure, arithmetic imputation using the nearest two weight measures, mixed-effects model with the lowest mean absolute error, generalized estimating equation (GEE) model with the lowest mean absolute error), Dar es Salaam, Tanzania, 2010–2013

For data visualization, we randomly selected eight individuals from the testing dataset of each study and plotted their observed weights and imputed weights based on the four methods (Figs. 1 and 2). For the mixed-effects model with the lowest MAE in each study, we further plotted the observed weight against the difference between the observed weight and the imputed weight at the target pregnancy time for the individuals included in the testing set (Supplement Figs. 3 and 4).

## Discussion

We compared four approaches to imputing early-pregnancy weight based on weights collected during pregnancy. This imputation procedure could be the first-stage analysis in an analysis with GWG as exposure or outcome. While the final goal may be to estimate a target parameter such as the association of GWG with a pregnancy outcome, if the imputed values resemble the underlying complete data closer, the estimates of the target parameter are more likely to be less biased and more efficient [28, 29]. Thus, our imputation models were compared based on the imputation error in this paper. We reported that the mixed-effects models had the highest overall imputation accuracy compared to the other three methods. We also found that mixed-effects models were robust for both the scenarios of extrapolation and interpolation based on the underlying distributions of available weights. The imputation error from the mixed-effects models could be as low as 1.6 to 2.0 kg, corresponding to approximately 3 to 4% of the average weight in early pregnancy. Comparing the results between the two studies, Study II with more participants and weight measurements, and earlier gestational age for the weight measurements, had more accurate imputation results. Specifically, comparing the MAEs between the interpolation on Study II and the extrapolation on Study I, we observed an approximate 20% lower in MAE for the mixed-effects model method, 30% lower for the GEE method, 30% lower for the simple arithmetic calculation, and 15% lower for the nearest weight measure assignment.

Overall, our results support the preferable use of mixed-effect models over GEE or more traditional approaches. When comparing the imputation errors between the two simple imputation approaches (i.e. assigning nearest weight and arithmetic imputation using nearest two weight measures) and the mixed-effects model approach, we saw a difference in MAEs up to 0.9 kg and 0.5 kg in weight extrapolation on Study I and weight interpolation on Study II, respectively. The relatively small differences in the imputation errors across the four methods may suggest that, compared to the simple arithmetic approaches, the use of mixed-effect models may not considerably impact the estimates in the epidemiological studies on gestational weight or GWG. However, modeling-based imputation, such as the mixed-effects model method, allows one to anchor the weight estimate at a specific time point of a pregnancy without making additional assumptions on the underlying gestational age distribution or the GWG trajectory for a given study. This is particularly important when there is heterogeneity in the gestational age at study baseline, the length of intervals between pregnancy measurements, or the trajectory of GWG across the study subjects. Since our study only evaluated the magnitude of differences across different imputation methods in imputing early-pregnancy weight, future studies are needed to further compare and quantify the differences in performance across different imputation methods at different time points of pregnancy.

In our study, we observed different patterns of imputation errors across the mixed-effects models with varying degree of model flexibility between weight extrapolation on Study I and weight interpolation on Study II. When extrapolating early-pregnancy weights with limited data available, our findings suggest that overfitting should be a concern when selecting the optimal mixed-effects model. When early-pregnancy weight data was not generally available (as in Study I), fewer knots or polynomial terms in mixed-effects models might outperform more complex models with additional model flexibility; dropping weights collected in later pregnancy might further improve accuracy. However, when interpolating early-pregnancy weight with earlier weights available in a study with a large sample size, allowing for model flexibility by adding additional splines or polynomial terms might slightly improve the model performance. Therefore, mixed-effects models with appropriate degrees of model flexibility based on the underlying study data structure should be considered when choosing the approach to impute early-pregnancy weight. In addition to MAE based on a testing set, the Akaike information criterion (AIC) and Bayesian information criterion (BIC), which do not require a testing set, can be used to compare different model choices in the spline terms.

Previous studies have attempted to impute missing pregnancy weight using different methods [7, 18–20, 26, 30, 31]. Most of the studies applied a simple arithmetic approach without using all the available weight measurements [7, 19, 20, 30, 31]. Our results suggested that having more weight data closer to the gestational week of interest then fitting models which allowed between-person variation would produce better imputation accuracy. Using weight data from a hospital-based study in the United States, Darling et al. evaluated performances between mixed-effects models and simple arithmetic methods for imputing week 28 and week 40 of gestation weight and reported similar findings (MAEs of 1.21–2.62 kg from their mixed-effects models) [26]. In this study, we imputed pregnancy weight at a different time of gestation, and the mixed-effects model still outperformed arithmetic imputation approaches, suggesting its potential application in imputing pregnancy weight at different time points. Similar to Darling et al., we found that adding covariates or variable transformation did not improve accuracy. Overall, the current literature suggests that the mixed-effects model can be a useful and robust approach to imputing pregnancy weight at different time points during pregnancy using repeated weight measures.

To our knowledge, this is the first study evaluating the GEE method in imputing pregnancy weight. Compared to the mixed-effects model method with random intercepts and slopes, the GEE method did not require any normality assumption and accounted for individual differences in GWG by adding a subject-specific residual to the group-level mean. This subject-specific residual was analogous to the random intercept in the mixed-effects model method. However, the GEE method did not take into account the between-subject variation in the slope of the time term in the regression model, while this was taken into account through random slopes in the mixed-effects model. In both studies, the GEE method performed poorly compared to the mixed-effects models, suggesting that including a subject-specific slope of the time term was necessary to capture the heterogeneity of GWG patterns among participants and that the robustness to normality in the GEE method did not compensate for the disadvantage of ignoring this subject-specific slope of the time term. Furthermore, the GEE method using the mean residual performed similarly to the nearest weight residual method for weight interpolation in Study II but outperformed the nearest weight residual method for weight extrapolation in Study I, indicating that different residual approaches should be considered when using the GEE method on datasets with different pregnancy weight distributions. Since the GEE method has rarely been used in previous studies, future studies should further evaluate its performance under different residual methods.

The imputation methods are valid under the missing at random (MAR) assumption which allows the probability of missingness depends on observed data [32]. Potential predictors of missingness probability should be considered in the imputation models. In both of our Studies I and II, the available weight measurements and gestational age were taken into account in the imputation model. When additionally including gravidity, age, and education status in the imputation models, the imputation accuracy was not improved. In an earlier study by Darling et al., similarly, adding covariates (i.e., age, height, gravidity, and gestational diabetes status) in the imputation model did not improve the level of accuracy [26]. This could be due to the fact that most information about the missing gestational weights was contained in the available weight measurements for the same individual due to the high within-person correlation over time for gestational weight, and after taking into account these available weight measurements, other variables may not contain much additional information about the missing weights. Future studies may continuously evaluate whether any other covariates could improve the imputation models. In addition, multiple imputation has been suggested as an alternative method to handle incomplete data under the case of MAR [33]. An analysis evaluating the association of GWG with pregnancy outcomes can use multiple imputation techniques so that the extra variation in the estimates of missing values can be taken into account in the interval estimates of the parameters of interest.

Our study had several strengths. First, we undertook imputation analyses on two separate data cohorts with repeated weight measurements, allowing us to evaluate the imputation performance under different availabilities of early-pregnancy weights. Second, we compared multiple traditional and novel imputation techniques, including the GEE method, with varying degrees of model flexibility. Given the importance of GWG on optimal pregnancy outcomes and the long-term health of mother and the offspring [3, 4, 6–9], our findings will benefit studies examining GWG with respect to pregnancy-related or future disease outcomes with limited weight measures, when the knowledge of early-pregnancy weight is critical to characterize GWG.

Our study had some limitations. First, there was no pre-pregnancy weight or body mass index available in either study, and only 15.7% of participants in Study I had first-trimester weights available. Given the availability of the data, we chose 14 weeks of gestation as the target point for weight imputation to avoid over-extrapolation. Consequently, we were unable to evaluate the imputation methods in imputing pre-pregnancy weight or pregnancy weight earlier than the target time point of 14 weeks of gestation. Nevertheless, the two studies that we used had different distributions of pregnancy weights, which represented imputing early-pregnancy weight under different scenarios. The consistent results between our two studies and the similar conclusions from the study by Darling et al. [26] suggested the robustness of the mixed-effects model approach in imputing pregnancy weight at different time points of pregnancy. Second, due to the limited number of women with early-pregnancy weights from Study I (*n* = 231), the size of the testing set was small. As a result, our results might have been influenced by a few extreme weight values. Furthermore, we did not have sufficient power to evaluate the imputation performance by creating multiple random testing sets to validate our findings. Last but not least, it is unclear whether our findings can be generalized to women outside of Tanzania or sub-Saharan Africa. However, the results on imputing pregnancy weights at week 14 and week 28 of gestation, based on a study of the predominantly Caucasian population in the United States had similar findings [26], supporting our conclusions on the robustness of the mixed-effects model approach.

## Conclusions

Our study suggests that mixed-effects models are useful in research settings to impute early-pregnancy weights when such measures were not available. Future studies are warranted to further validate the mixed-effects model approach in other studies and in imputing pregnancy weights at different time points of pregnancy. The utility of GEE and multiple imputation approaches should also be further investigated in future work.

## Supplementary Information

**Additional file 1: Supplement Figure 1.** Observed pregnancy weights (kg) of 20 randomly selected subjects from Study I, Dar es Salaam, Tanzania, 2010–2012.

**Additional file 2: Supplement Figure 2.** Observed pregnancy weights (kg) of 20 randomly selected subjects from Study II, Dar es Salaam, Tanzania, 2010–2013.

**Additional file 3: Supplement Figure 3.** Observed weight versus the difference between the observed and imputed weights, for 200 subjects included in Study I testing set based on the mixed-effects model with the lowest mean absolute error (kg), Dar es Salaam, Tanzania, 2010–2012. The upper 95% limit was calculated by adding two standard deviations of the differences to the mean difference; the lower 95% limit was calculated by subtracting two standard deviations of the differences from the mean difference. The majority of the plotted subjects fall within the lower and upper limits, suggesting a good agreement between the observed and imputed weights.

**Additional file 4: Supplement Figure 4.** Observed weight versus the difference between the observed and imputed weights, for 200 subjects included in Study II testing set based on the mixed effects model with the lowest mean absolute error (kg), Dar es Salaam, Tanzania, 2010–2013. The upper 95% limit was calculated by adding two standard deviations of the differences to the mean difference; the lower 95% limit was calculated by subtracting two standard deviations of the differences from the mean difference. The majority of the plotted subjects fall within the lower and upper limits, suggesting a good agreement between the observed and imputed weights.

## Data Availability

The datasets analyzed during the current study are not publicly available due to regulatory obligations of the collaborating institutions but are available from the corresponding author on reasonable request.

## Abbreviations

GWG: Gestational weight gain
GEE: Generalized estimating equation
MAE: Mean absolute error
MSE: Mean square error
