# Supplementary figures and images for: Methodological approaches to imputing early-pregnancy weight based on weight measures collected during pregnancy
Source: BMC Med Res Methodol. 2021 Feb 5;21:24. doi: 10.1186/s12874-021-01210-3 (PMC7863454; doi:10.1186/s12874-021-01210-3)

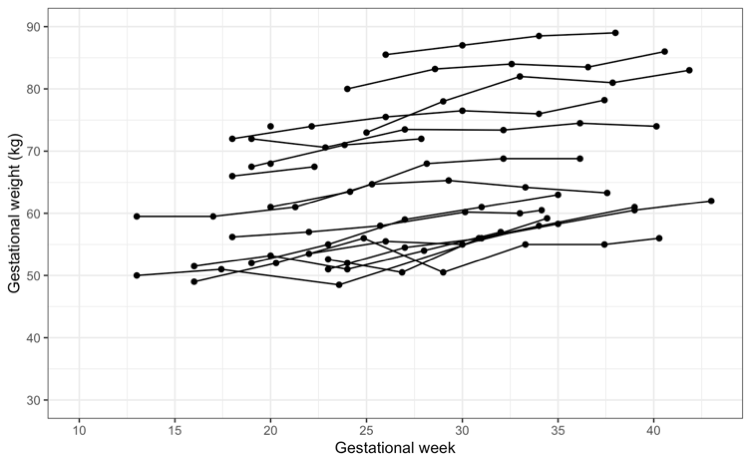

Supplement: Supplementary file 1 — Additional file 1: Supplement Figure 1. Observed pregnancy weights (kg) of 20 randomly selected subjects from Study I, Dar es Salaam, Tanzania, 2010–2012. [file 12874_2021_1210_MOESM1_ESM.png]

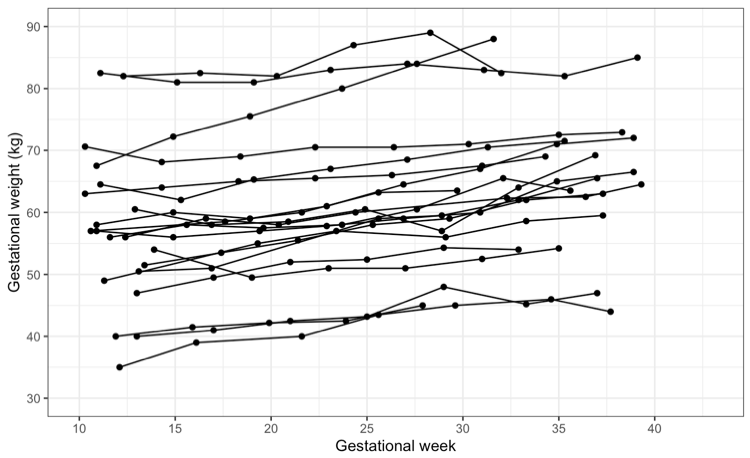

Supplement: Supplementary file 2 — Additional file 2: Supplement Figure 2. Observed pregnancy weights (kg) of 20 randomly selected subjects from Study II, Dar es Salaam, Tanzania, 2010–2013. [file 12874_2021_1210_MOESM2_ESM.png]

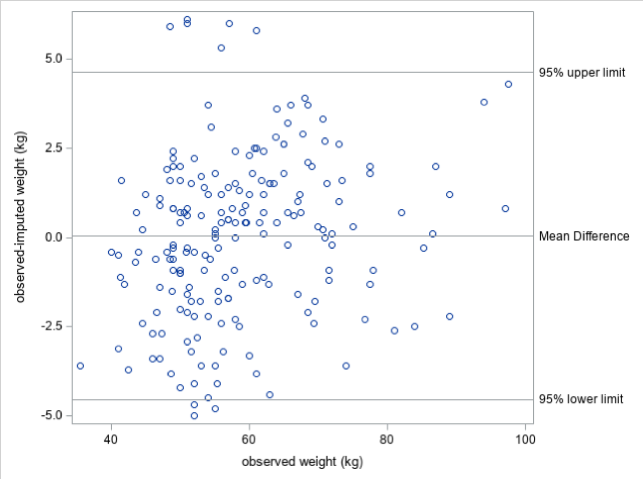

Supplement: Supplementary file 3 — Additional file 3: Supplement Figure 3. Observed weight versus the difference between the observed and imputed weights, for 200 subjects included in Study I testing set based on the mixed-effects model with the lowest mean absolute error (kg), Dar es Salaam, Tanzania, 2010–2012. The upper 95% limit was calculated by adding two standard deviations of the differences to the mean difference; the lower 95% limit was calculated by subtracting two standard deviations of the differences from the mean difference. The majority of the plotted subjects fall within the lower and upper limits, suggesting a good agreement between the observed and imputed weights. [file 12874_2021_1210_MOESM3_ESM.png]

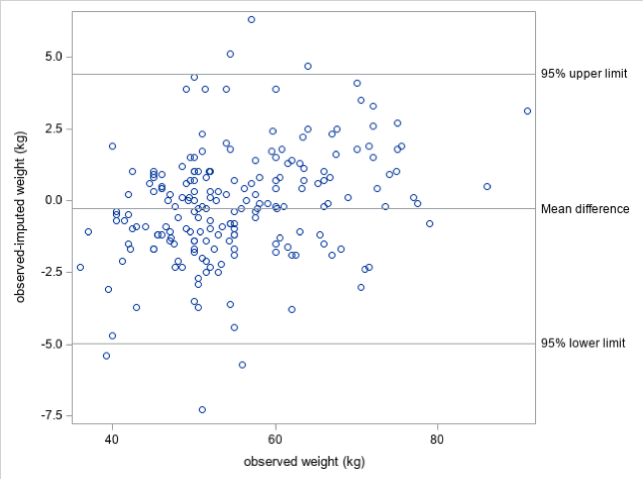

Supplement: Supplementary file 4 — Additional file 4: Supplement Figure 4. Observed weight versus the difference between the observed and imputed weights, for 200 subjects included in Study II testing set based on the mixed effects model with the lowest mean absolute error (kg), Dar es Salaam, Tanzania, 2010–2013. The upper 95% limit was calculated by adding two standard deviations of the differences to the mean difference; the lower 95% limit was calculated by subtracting two standard deviations of the differences from the mean difference. The majority of the plotted subjects fall within the lower and upper limits, suggesting a good agreement between the observed and imputed weights. [file 12874_2021_1210_MOESM4_ESM.png]
